# Long-term plasticity determines the postsynaptic response to correlated afferents with multivesicular short-term synaptic depression

**DOI:** 10.1186/1471-2202-15-S1-P61

**Published:** 2014-07-21

**Authors:** Alex D Bird, Magnus JE Richardson

**Affiliations:** 1 Warwick Systems Biology Centre, University of Warwick, Coventry CV4 7AL, UK; 2 Warwick Systems Biology DTC, University of Warwick, Coventry CV4 7AL, UK; 3 School of Life Sciences, University of Warwick, Coventry CV4 7AL, UK

## 

The firing rate of a neuron is largely determined by correlations in synaptic drive. Correlations in neurotransmitter release between different active sites arise from both synchronous activity in the presynaptic population and the number of independent release sites per neuron. Recent work has shown that the number of release sites on each neuron is modified during long-term plasticity [1]. Such changes will modulate the effect of synchronous drive and therefore have a significant effect on the response of the postsynaptic cell.

To understand how correlations from synaptic dynamics and from presynaptic synchrony shape the postsynaptic response, we studied a model of multiple release site short-term plasticity and derived exact results for the crosscorrelation function of vesicle occupancy and neurotransmitter release, as well as the postsynaptic voltage variance [2]. Using approximate forms for the postsynaptic firing rate in the limits of low and high correlations, we demonstrated that short-term depression leads to a maximum response for an intermediate number of presynaptic release sites, and that this leads to a tuning-curve response peaked at an optimal presynaptic synchrony set by the number of independent neurotransmitter release sites per presynaptic neuron (Figure 1).

**Figure 1:**
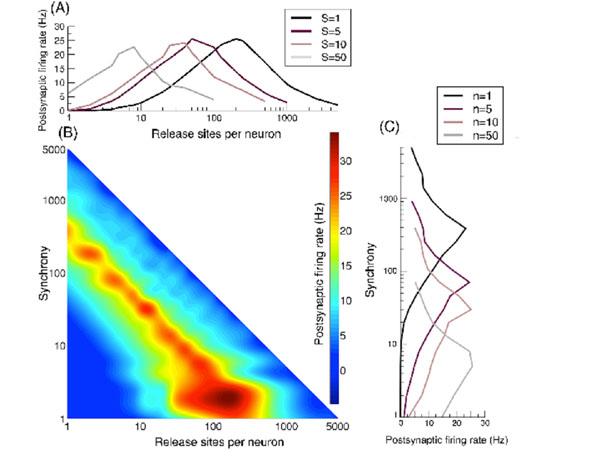
**Long-term plasticity, which alters release-site number *n*, sets the sensitivity to presynaptic synchrony *S*. (A)** Postsynaptic rate as a function of release-sites per presynaptic neuron *n* for different numbers of presynaptic cells firing together *S* (synchrony). **(B)** Heat map of the postsynaptic rate as a function of presynaptic release-site number *n* and presynaptic synchrony *S*. **(C)** Postsynaptic rate as a function of presynaptic synchrony *S* for different examples of release-site number. Long-term potentiation makes the postsynaptic cell more sensitive to weak synchrony, whereas long-term depression sensitizes the cell to stronger synchrony.

These effects arise because, above a certain level of correlation, activity in the presynaptic population is overly strong resulting in wastage of the pool of releasable neurotransmitter. As the nervous system operates under metabolic constraints it is likely that this phenomenon provides an activity-dependent constraint on network architecture.

## References

[B1] Loebel A Le Be JV Richardson MJE Markram H Herz AVM “Matched Pre- and Post-Synaptic Changes Underlie Synaptic Plasticity over Long Time Scales,” J Neurosci 2013 15 6257 6266 10.1523/JNEUROSCI.3740-12.2013 23575825 PMC6619066

[B2] Bird AD Richardson MJE “Long-term plasticity determines the postsynaptic response to correlated afferents with multivesicular short-term synaptic depression,” Front Comput Neurosci 2014 15 Article 2 24523691 10.3389/fncom.2014.00002 PMC3906582

